# Editorial: Application and evaluation of acupuncture in the treatment of neurological diseases

**DOI:** 10.3389/fneur.2023.1201234

**Published:** 2023-06-01

**Authors:** Chen Chen, Liming Lu, Chunzhi Tang, Myeong Soo Lee, Nenggui Xu

**Affiliations:** ^1^Clinical Research and Big Data Laboratory, South China Research Center for Acupuncture and Moxibustion, Medical College of Acu-Moxi and Rehabilitation, Guangzhou University of Chinese Medicine, Guangzhou, Guangdong Province, China; ^2^KM Science Research Division, Korea Institute of Oriental Medicine, Daejeon, Republic of Korea

**Keywords:** acupuncture, clinical studies, evidence integration, evidence-based medicine, interdisciplinary technologies, clinical evidence, clinical research

Neurological disorders are a significant cause of disability and death worldwide, and they have been posing a serious burden on global health systems, particularly in low- and middle-income countries for the past 35 years (1). Acupuncture, the most widely used traditional Chinese medical practice globally, has been used in 183 countries and regions, and it is a key treatment option for neurological diseases (2).

This Research Topic entitled “*Application and evaluation of acupuncture in the treatment of neurological diseases*” consists of 27 manuscripts focusing on neurological diseases such as Stroke, Pain syndromes, Parkinson's disease (PD), Substance use disorders, Bell's palsy (BP), etc. These contributions offer interesting insights into the effectiveness and safety of acupuncture for the treatment of neurological disorders.

## 1. Stroke

This specific issue includes six studies focusing on stroke. Stroke is a condition with increasing incidence and prevalence, leading to a significant socio-economic burden and loss of healthy life years worldwide (3). Acupuncture, as a practical and safe traditional Chinese medicine treatment, has been widely used in the rehabilitation of stroke patients (4).

Li, Zhu et al. conducted a multicenter, randomized, parallel controlled trial to evaluate the effectiveness of acupuncture treatment for ischemic stroke rehabilitation. A total of 497 patients with ischemic stroke were enrolled, and the results showed that acupuncture treatment had a better recovery effect than rehabilitation alone.

In the second study by Kim et al., the authors conducted a double-blind, randomized controlled trial (RCT), recruiting 33 patients with subacute stroke. The control group received conventional rehabilitation treatment, while the experimental group received 30 min of electroacupuncture in addition to rehabilitation. The results of the study supported that electroacupuncture could be an effective adjunctive treatment for motor recovery after stroke.

Motor aphasia is one of the most common sequelae after stroke. In a network meta-analysis study by Feng et al., it was found that scalp-tongue acupuncture combined with speech training may be the best acupuncture-related therapy to improve clinical outcomes in patients with motor aphasia after stroke.

Spasticity is also one of the most common complications after stroke, with a prevalence of 30–80% (5). Acupuncture had a better effect than conventional treatment in relieving post-stroke spasticity and could be recommended as an adjunctive treatment for spasticity after stroke. This is confirmed by Xue et al. in their systematic review and meta-analysis of 88 studies.

Disorders of consciousness are prevalent among stroke patients. According to a meta-analysis by Huang Z. et al. acupuncture was found to be more effective in enhancing the level of consciousness, increasing resuscitation rates, and reducing resuscitation times compared to patients who did not receive acupuncture treatment.

Also in this issue, Choi et al. conducted a comprehensive review of 48 published systematic reviews on acupuncture interventions for stroke rehabilitation. The results suggest that acupuncture could potentially be a safer and more effective alternative to rehabilitation in the management of post-stroke shoulder-hand syndrome.

## 2. Parkinson's disease

PD affects 8.5–10 million people globally, and its prevalence increases with age (6). Qi Huang Needle (QHN) therapy, a combination of acupuncture and manipulation, was used to treat PD in a RCT conducted by Li, Zhou et al. They concluded that QHN therapy was consistently superior to sham acupuncture in reducing both motor and non-motor symptoms and significantly improving muscle stiffness in PD patients.

## 3. Pain syndromes

Acupuncture is a widely used method for alleviating both acute and chronic pain (7, 8). Kang et al. identify an important issue about tension-type headaches (TTH) by reviewing 31 RCTs. They concluded that acupuncture may be a safe and effective treatment for patients suffering from TTH. In a separate study, Liu et al. investigated the impact of caffeinated beverage intake on the analgesic effect of electroacupuncture in a randomized controlled trial. The study provides evidence for possible reasons for differences in acupuncture effectiveness between Western and Eastern populations.

## 4. Substance use disorders

Patients who are receiving methadone maintenance treatment (MMT) for opioid use disorder often experience several challenges such as sleep disturbances and headaches (9). Dong et al. had looked at the effects of acupuncture assisted MMT therapy by using a decision tree model. They observed that acupuncture can aid patients in achieving a more proficient reduction of methadone by alleviating the aforementioned side effects.

## 5. Bell's palsy

BP is a common peripheral facial palsy, accounting for 70% of all cranial mono-neuropathies (10). Acupuncture is commonly used as a complementary therapy for Bell's palsy, however, there is still a debate on the timing of its initiation. In this regard, the papers by Yang et al. and Lan et al. contribute to this aspect. The first one is a retrospective study which showed that acupuncture intervention during the acute stage of BP could shorten recovery time and improve outcomes. In the other paper, the authors presented a clinical case report of a 27-year-old pregnant patient diagnosed with BP, which was treated successfully with five courses of acupuncture treatment resulting in complete recovery.

## 6. Other neurological diseases

Lee et al. summarized the literature on the efficacy of acupuncture in treating overactive bladder (OAB) symptoms. The results showed that acupuncture had a more favorable effect than sham acupuncture in reducing the symptoms of OAB. Furthermore, acupuncture was found to be as effective as conventional medication in improving OAB symptoms.

Huang Y. et al. performed a comprehensive quantitative and qualitative analysis of publications related to acupuncture for spinal cord injury (SCI). The study found that China and the United States were the hub countries for related publications. Additionally, the authors predicted that there will be more studies on electroacupuncture for promoting nerve repair and regeneration after SCI in the future.

Zhang et al. explored brain activity and neural mechanisms after acupuncture using functional magnetic resonance imaging (fMRI). The study found that acupuncture stimulation can effectively relieve the severity of tinnitus by decreasing functional connectivity of amygdala in subjective tinnitus patients. Huang H. et al. conducted a meta-analysis to study brain activity after acupuncture. The results showed that acupuncturing on ST36 could positively activates the opercular part of the right inferior frontal gyrus (IFG.R), left superior temporal gyrus (STG.L), and right median cingulate/paracingulate gyri (MCG.R) regions. In two other studies (Chen et al.; Li, Lai et al.), researchers examined the best treatment options for acupuncture in the treatment of tic disorders as well as mild cognitive impairment. These studies will serve as a good reference for researchers interested in the acupoint selection and the use of fMRI techniques in acupuncture treatment.

Apart from the aforementioned case report involving BP, we also received four other case reports. These reports included a 50-year-old woman with hemifacial spasm and temporomandibular joint pain (Huang J.-p. et al.); a woman and a man each with olfactory dysfunction after COVID-19 (Morita et al.); a woman with Guillain-Barré syndrome (Li, Xu et al.); and a child in a persistent vegetative state after herpes simplex virus encephalitis (Jin et al.). All these patients achieved satisfactory therapeutic results after receiving acupuncture treatment.

## 7. Afterword

In conclusion, we hope that the above studies provide compelling evidence for the effectiveness and safety of acupuncture in treating neurological diseases. It is our hope that this evidence will accelerate the incorporation of acupuncture into clinical practice for the treatment of neurological diseases.

## Author contributions

CC and LL contributed equally to the writing and editing of this manuscript. All authors contributed to the article and approved the submitted version.
